# Fluorometric toolkit for methanethiol quantification and methanethiol oxidase activity determination in biological systems

**DOI:** 10.1016/j.redox.2026.104288

**Published:** 2026-07-08

**Authors:** Thilo M. Philipp, Belen Schiffmann, Lucie K. Tintrop, Jacqueline Findlay, Niklas Krafczyk, Hanna Schlemminger, Susana Ainsa-Zazurca, Vicente Ferreira, Pascal Fuchsmann, Patrice Nordmann, Lars-Oliver Klotz, Csaba Szabo, Tomas Majtan

**Affiliations:** a Pharmacology Unit, Department of Oncology, Microbiology and Immunology, Faculty of Sciences and Medicine, University of Fribourg, Fribourg, Switzerland; b Agroscope, Food Microbial Systems Research Division, Bern, Switzerland; c Microbiology Unit, Department of Oncology, Microbiology and Immunology, Faculty of Sciences and Medicine, University of Fribourg, Fribourg, Switzerland; d Institute of Nutritional Sciences, Nutrigenomics Section, Friedrich Schiller University Jena, Jena, Germany; e Laboratorio de Análisis del Aroma y Enología, Departmento de Química Analítica, Facultad de Ciencias, Instituto Agroalimentario de Aragón-IA2, Universidad de Zaragoza-CITA, Zaragoza, Spain

**Keywords:** Methanethiol, Volatile sulfur compound, Methanethiol oxidase, Assay development

## Abstract

Methanethiol (MeSH) is a pivotal volatile sulfur compound in plant, human, and environmental chemistry, but its study is hindered by analytical challenges. Here, we present an accessible fluorometric two-component assay toolkit for quantitative assessment of its content and enzymatic degradation by methanethiol oxidase (MTO). The first assay sensitively detects MeSH with a limit of detection of 124 nM using a novel “tube-in-tube” design for efficient MeSH capture, circumventing the need for complex chromatography and expensive instrumentation. The second assay is a real-time kinetic MTO activity assay. Using this toolkit, we (i) established an inverse correlation between MTO activity and MeSH levels in a colon cancer cell line, (ii) quantified MeSH as a key scent marker in various types of cheese, (iii) evaluated MeSH release from the selected *Brassica* and *Allium* species and (iv) determined its production in aquatic systems. The MeSH metabolism toolkit presented here will provide researchers with simple, sensitive and specific assays to elucidate multifaceted roles of MeSH and MTO in human pathophysiology and to accelerate discoveries in a broad spectrum of scientific disciplines.

## Highlights

• Fluorometric tube-in-tube assay quantifies MeSH with 124 nM LOD.
• Real-time kinetic assay measures MTO activity via H_2_S and H_2_O_2_ products.
• New MTO assay is up to 60-fold more sensitive than endpoint assays.
• MTO activity inversely correlates with MeSH in colorectal cancer cells.
• Provided toolkit quantifies MeSH in diverse biological samples.

## Abbreviations

AzMC: 7-Azido-4-methylcoumarin
BSA: Bovines serum albumin
CBA: coumarin boronic acid
CH_3_S^−^: Methanethiol anion
CH_3_SNa: Sodium methanethiolate
DBD-F: 4-(N,N-Dimethylaminosulfonyl)-7-fluoro-2,1,3-benzoxadiazole
DMDS: Dimethyldisulfide
GC/SCD: gas chromatography with a sulfur chemiluminescence detector
H_2_S: Hydrogen sulfide
LOD: Limit of detection
LOQ: Limit of quantification
MeSH: Methanethiol
MGL: Methionine γ-lyase
MTO: Methanethiol oxidase
NaOH: Sodium hydroxide
Ppb/ppt: Parts per billion/trillion
R^2^: correlation coefficient
TCEP: Tris(2-carboxyethyl)phosphine
VOC: Volatile organic compounds
VSC: Volatile sulfur compounds

## 1 Introduction

**Fig. 1:**
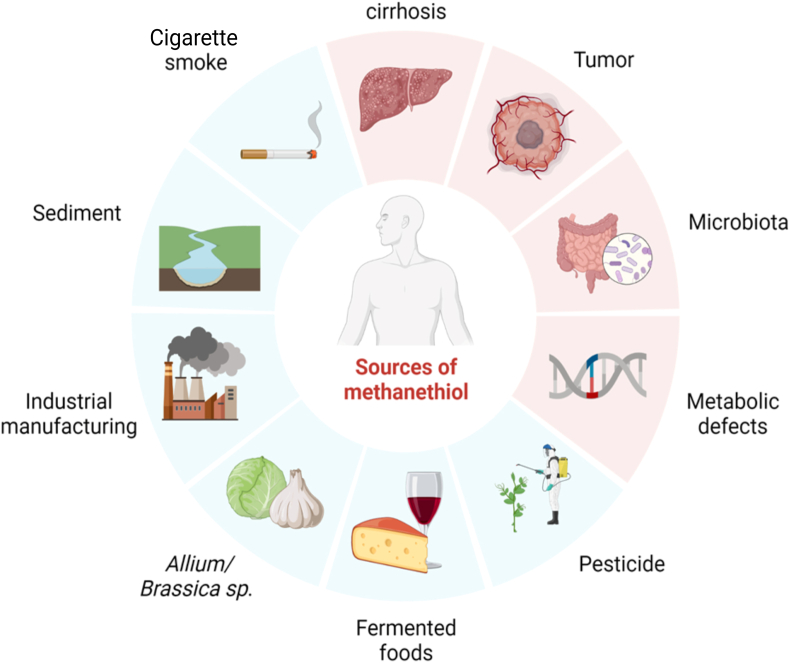
**Potential sources of methanethiol.** Blue and red colors indicate exogenous and endogenous sources of human exposure, respectively.

Methanethiol (CH_3_SH or MeSH), a highly toxic volatile sulfur compound (VSC), is garnering increasing interest across diverse scientific fields, yet its study is hindered by significant analytical challenges (Fig. 1) [1]. In plants, MeSH serves a multitude of functions; it is a key component of sulfur metabolism and serves as a signaling molecule that mediates plant-pathogen interactions, root-microbiome communication, and pollinator attraction [[2], [3], [4], [5]]. Detecting MeSH in soil headspace, the rhizosphere, or plant emissions could therefore provide real-time insights into physiological stress responses. Concurrently, an increased interest in MeSH and metabolism of VSCs has recently emerged in the fields of inborn errors of metabolism and oncology. Defects in the methionine cycle and transsulfuration pathway as well as impaired degradation of MeSH due to methanethiol oxidase (MTO) dysfunction, create metabolic bottlenecks that elevate MeSH levels in affected patients, manifesting in extraoral halitosis [[6], [7], [8]]. Furthermore, MeSH is considered a potential volatile scent marker of cancer. In food chemistry, MeSH is a dual-purpose molecule, serving as a key aroma compound in certain contexts while acting as a marker of spoilage in others [9,10]. Additionally, MeSH has recently gained particular interest in environmental sciences due to its role in cloud formation and regulation of Earth's temperature (the CLAW hypothesis) [11,12].

Despite this broad and compelling relevance, the accurate, sensitive, and accessible detection of MeSH remains a formidable analytical challenge as a direct consequence of its intrinsic chemical properties. The very characteristics that render MeSH biologically active also make it a difficult analyte. Its low molecular weight (48 g/mol), high vapor pressure, and relatively high acid dissociation constant (pKa ≈10.4) promote rapid volatilization, leading to significant sample loss [13]. Its potent nucleophilicity, driven by a polarizable sulfur atom, makes it highly reactive [14]. It has been shown that MeSH oxidizes to dimethyl disulfide (DMDS) in the presence of oxygen or trace metals and can form complexes with metal ions or adsorb irreversibly to common laboratory surfaces [15,16]. These attributes, combined with its typically low part-per-billion (ppb) to part-per-trillion (ppt) physiological concentration, create numerous analytical difficulties. Current state-of-the-art methodologies, such as gas chromatography coupled with sulfur-selective or mass spectrometry detectors, require specialized operational expertise, expensive, non-portable equipment coupled with a complex, time-consuming sample preparation and thus are ill-suited for practical, routine research [13]. This creates a significant hurdle practically preventing many life sciences researchers from incorporating MeSH analysis into their projects, such as measuring MeSH in tissues and cells or monitoring MTO enzymatic activity. However, despite these diverse biological and environmental roles, no existing analytical platform offers a rapid, sensitive, and user-friendly method to quantify methanethiol across such heterogeneous matrices.

To fill this critical gap and enable research in metabolism of MeSH and VSCs, here we present a novel methodological two-component toolkit for a highly sensitive, selective and straightforward fluorometric quantification of trace amounts of MeSH and for assessing the degradation of MeSH by MTO.

## 2 Results

### 2.1 Novel fluorometric MeSH quantification assay

**Fig. 2:**
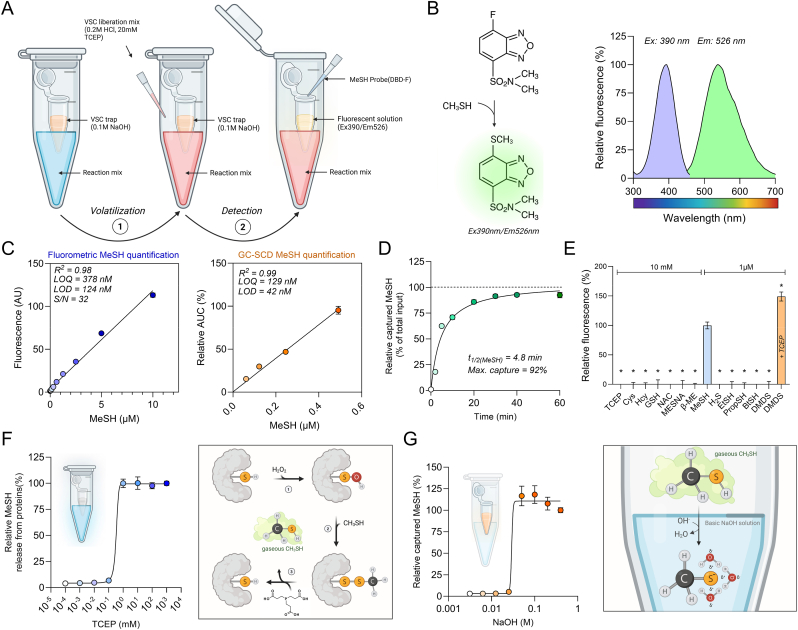
**Design and development of the novel fluorometric MeSH quantification assay. A** – Schematic assay workflow illustrating the volatilization and trapping of MeSH. **B** – Reaction scheme for the derivatization of MeSH (10 μM) with the fluorogenic probe DBD-F (10 μM) and the resulting spectral properties of the product (λ_ex_/λ_em_-spectra were recorded at fixed 526nm/390 nm, respectively). **C** – Standard curves for MeSH quantification were generated using our fluorogenic assay (n = 8) and the state-of-the-art GC-SCD method (n = 2) for comparison. The limit of detection (LOD), limit of quantification (LOQ) and signal-to-noise ratio (S/N) for MeSH concentrations around the LOQ are indicated. **D** – Time-dependent capture and loss of MeSH during phase transfer. MeSH standard (10 μM) was subjected to a phase transfer over a period of 60 min, followed by derivatization with DBD-F at designated time points. As a reference for maximum recovery, an equivalent MeSH standard was directly derivatized with DBD-F without undergoing a phase transfer. The relative amount of captured MeSH was calculated as a percentage ratio of trapped MeSH to directly derivatized MeSH (n = 8). **E** – Assay selectivity showing the fluorescence response to MeSH compared to common biological thiols and other alkylmercaptans after 1 h of a phase transfer (n = 5). **F** – Optimization of TCEP concentration for the release of disulfide-bound MeSH from BSA (n = 8). Methanethiolated BSA (100 μl) was incubated with an equal volume of increasing concentrations of TCEP. After the phase transfer and capture, the released MeSH was derivatized with DBD-F. The scheme on the right illustrates the underlying chemistry: oxidation of protein sulfhydryl residues generates reactive intermediates, which react with MeSH to form mixed disulfides. These disulfide bonds can be cleaved by a reducing agent, such as TCEP, liberating free MeSH for detection. **G** – Optimization of NaOH concentration as a VSC trapping solution (n = 8). MeSH (10 μM) was volatilized and subjected to a phase transfer with the gaseous MeSH trapped using 100 μl of NaOH solutions at varying concentrations. The scheme on the right illustrates the trapping mechanism: in alkaline solutions, MeSH is deprotonated to the non-volatile methanethiolate anion (CH_3_S^−^), which is retained in the aqueous trap and subsequently derivatized with DBD-F. All data are presented as mean ± SEM and asterisk (*) indicates significance (p < 0.05).

To establish a minimalistic, in-house assay for quantifying MeSH, we adapted the fluorogenic derivatization principle of Toda et al. into a streamlined, solution-based protocol (Fig. 2A) [17]. Unlike the approach by Toda et al. using a three-stage process (gas absorption in a microchannel scrubber, fluorescent derivatization in a heated reactor and signal measurement via fluorometric detection), our method employs a simple “tube-in-tube” design, where an inner tube containing an alkaline solution (“VSC-trap”) captures gaseous MeSH and VSCs generated by the sample reaction mixture in an outer tube (Fig. 2A). The optional reduction and acidification step results in quantification of total MeSH by converting all sources, including the oxidized (DMDS) and protein-bound MeSH, into a volatile form that can be detected [18]. Following its separation from other non-volatile thiols, MeSH is thus selectively reacted with a fluorogenic probe 4-(N,N-dimethylaminosulfonyl)-7-fluoro-2,1,3-benzoxadiazole (DBD-F) yielding 4-(N,N-dimethylaminosulfonyl)-7-methylsulfanyl-2,1,3-benzoxadiazole (DBD-SCH_3_) as confirmed by ^1^H NMR (Fig. 2B, Supplementary Fig. 1), distinguishing it from other volatile organic compounds (VOCs). Importantly, this entire setup requires only a standard benchtop fluorometer or a fluorescence microplate reader, which are ubiquitous in modern life sciences laboratories. The assay exhibited a strong linear response across a range of 0-10 μM MeSH with a correlation coefficient (R^2^) of 0.98 (Fig. 2C–Supplementary Table 1). The limit of detection (LOD) and limit of quantification (LOQ) were calculated to be 124 nM and 378 nM, respectively. After the final assay optimization, the signal-to-noise ratio was determined to be > 32 for MeSH concentrations around the LOQ, indicating a robust and readily detectable signal. This sensitivity is comparable to the state-of-the-art method using gas chromatography equipped with a sulfur chemiluminescence detector (GC/SCD), which reported an LOD and LOQ of 42 nM and 129 nM, respectively (Fig. 2C–Supplementary Table 2).

Overall, volatilization and phase transfer represent a rapid and efficient process, capturing approximately 92% of MeSH after 30 min (Fig. 2D). The specificity and selectivity of our newly developed MeSH quantification assay were evaluated by using various thiol-containing compounds commonly found in biological samples and structurally related alkylmercaptans (Fig. 2E). Although DBD-F derivatizes thiols upon direct reaction (Supplementary Fig. 2), most of the tested thiols cannot undergo phase transfer in our assay. Common biological thiols, such as cysteine and glutathione, produced a negligible fluorescence signal at physiologically relevant concentrations, due to their lack of volatility. Furthermore, hydrogen sulfide (H_2_S) and alkylmercaptans with increasing carbon chain lengths (e.g., ethanethiol, propanethiol) did not significantly interfere with MeSH quantification within its expected analytical range (Fig. 2E). Meaningful interference was only observed when the system was overloaded with a 100-fold excess of these competing alkylmercaptans, a condition unlikely to be encountered in practical applications (data not shown). The fact that DMDS is only detected following reduction using Tris(2-carboxyethyl)phosphine (TCEP) underscores the assay's specificity for the free thiol functional group, as the disulfide bond itself is not reactive.

Further, we optimized the volatilization step required to quantify total MeSH in complex biological samples. First, we generated a model substrate using MeSH-conjugated bovine serum albumin (BSA). This was achieved by first oxidizing the cysteine residues of BSA to sulfenic acid intermediates using hydrogen peroxide (H_2_O_2_), which were then reacted with MeSH to form mixed disulfide bonds (Fig. 2F). Titration with a reductant TCEP demonstrated that a concentration of 1 mM TCEP was sufficient to liberate 100% of the bound MeSH without interference on the subsequent derivatization step (Fig. 2F). We also optimized quantitative capture of the volatilized MeSH. The basic sodium hydroxide (NaOH) solution, providing hydroxide ions (OH^−^), deprotonates MeSH in an acid-base reaction, forming the methanethiolate anion (CH_3_S^−^) and water. The negatively charged methanethiolate ion attracts the partially positive hydrogen ends of surrounding water molecules, forming a stable hydration shell that effectively traps the species within the solution. The efficiency of MeSH capture into the derivatization solution is dependent on NaOH concentration. Our results indicate that a 100 mM NaOH solution was necessary and sufficient for quantitative capture of the MeSH (Fig. 2G). While increasing NaOH concentration from 100 mM to 400 mM did not improve MeSH capture efficiency, it resulted in a ∼20% reduction in the final fluorescence signal.

### 2.2 Real-time kinetic MTO enzymatic assay

**Fig. 3:**
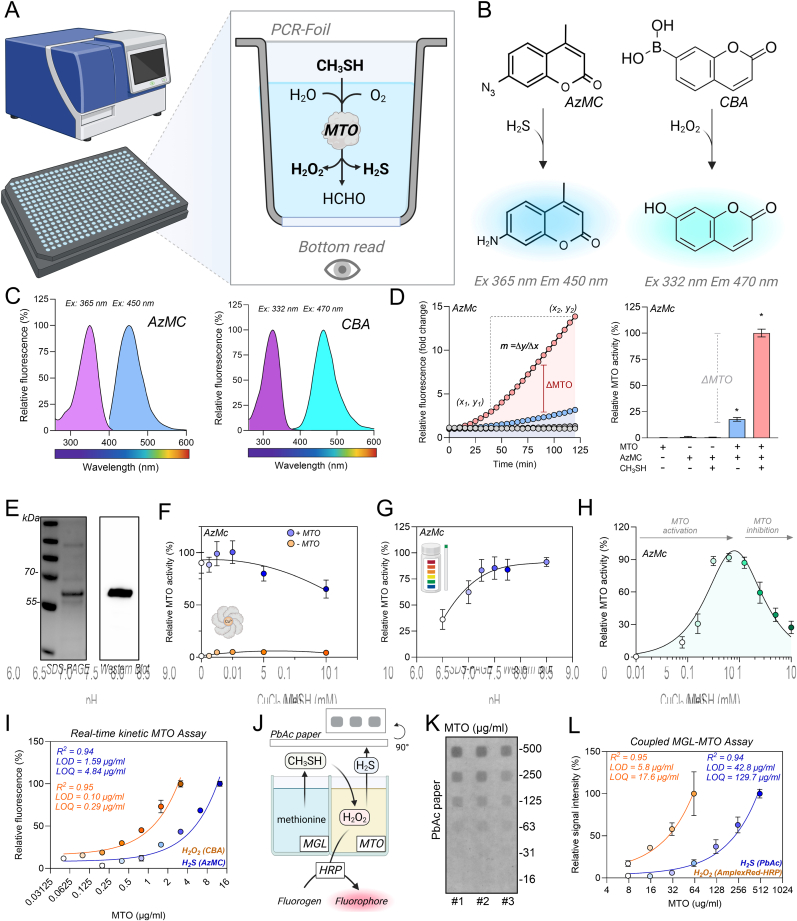
**Development of the real-time kinetic MTO enzymatic assay. A** – Schematic depiction of the MTO activity assay setup. **B** – Reaction schemes for the fluorometric detection of MTO products H_2_S by AzMC and H_2_O_2_ by CBA. **C** – Normalized spectral properties of the fluorescence probes after derivatization. AzMC (10 μM) was reacted with 10 μM Na_2_S, and CBA (10 μM) was reacted with 10 μM H_2_O_2_. The excitation and emission spectra were recorded at fixed emission wavelength of 450 nm and fixed excitation wavelength of 365 nm for AzMC and fixed emission wavelength of 470 nm and fixed excitation wavelength of 332 nm for CBA. **D** – Representative kinetic traces of MTO activity in cell lysates of MTO wildtype expressing HCT116 cells (H_2_S/AzMC). The left panel illustrates the calculation of MTO activity from the initial velocity (slope), while the right panel shows the final quantified data of the color-matched groups (n = 12). **E** – Purification of the recombinant MTO assessed by SDS-PAGE and Coomassie staining (left) and Western blot (right) probed with anti-MTO antibody. **F –** The effect of copper supplementation on MTO activity of purified MTO (n = 8). Recombinant MTO (1 μg) was subjected to the standard assay procedure with increasing concentrations of CuCl_2_ added to the reaction mixture (H_2_S/AzMC). **G** – pH dependence of MTO activity. Recombinant MTO (1 μg) was subjected to a modified version of the MTO assay in which HBS buffer was replaced with 100 mM Bis-Tris buffer at various pH values (H_2_S/AzMC). **H** – MTO enzyme kinetics showing substrate inhibition (n = 8). Recombinant MTO was used in the MTO assay with MeSH substrate concentrations ranging from 0.1 to 10 mM (H_2_S/AzMC). **I** – Assessment of the real-time kinetic MTO enzymatic assay sensitivity using H_2_S/AzMC and H_2_O_2_/CBA detection. Serial dilutions of recombinant MTO at different concentrations were used in the MTO assay to determine the LOD and LOQ. **J** – Schematic principle of the coupled MGL-MTO assay (left) and the example of its H_2_S/PbAc paper readout (right). **K** – Coupled MGL-MTO assay sensitivity using H_2_S/PbAc showing three technical replicates. **L** – Assessment of the coupled MGL-MTO assay sensitivity using H_2_S/PbAc and H_2_O_2_/HRP detection for comparative purposes with the data shown in panel **I**. All data are presented as mean ± SEM and asterisk (*) indicates significance (*p* < 0.05).

The second component of our MeSH metabolism toolkit is a sensitive, continuous MTO activity assay. This assay reports on MTO activity in a real-time kinetic fashion by quantifying products of MeSH degradation H_2_S and H_2_O_2_, while avoiding loss of volatile components in a closed system (Fig. 3A). The enzymatic reaction is initiated by combining an MTO-containing sample with MeSH trapped in an alkaline solution. MTO activity is continuously monitored by detecting H_2_S and H_2_O_2_ using the fluorescent probes 7-Azido-4-methylcoumarin (AzMC, Ex/Em: 365/450 nm) and coumarin boronic acid (CBA, Ex/Em: 332/470 nm), respectively (Fig. 3B). Due to spectral overlaps, these measurements are performed in separate wells (Fig. 3C). Further, we evaluated key enzymatic assay considerations and parameters using the H_2_S/AzMC approach as an example. After subtracting the fluorescent signal in the absence of MeSH, the enzyme activity is calculated from the initial velocity (the slope of the linear phase of the fluorescent signal) (Fig. 3D). To establish and investigate critical parameters of this method, we used recombinant MTO generated as described previously (Fig. 3E) [19]. Titration with copper chloride indicated that the as-purified MTO was essentially saturated with the catalytic copper cofactor, while the copper concentrations above 5 μM (corresponding to a Cu:MTO ratio of 22:1 in this setup) suppressed the measured activity (Fig. 3F). As the enzyme retained substantial activity without added copper in the assay buffer, and to avoid interference, copper was omitted from the standard assay conditions. The pH profile of MTO activity assay was determined using Bis-Tris buffer across a pH range of 6.5 to 8.5. Maximum fluorescence signal was observed at the pH 7.5-8.5 (Fig. 3G). Thus, 100 mM HEPES at pH 8.0 was selected in the final assay setup. Enzyme kinetics characterization showed that MTO follows classical Michaelis-Menten kinetics at low substrate concentrations but displayed pronounced substrate inhibition at higher MeSH concentrations (Fig. 3H). The calculated apparent K_m_ was 120 ± 50 μM with substrate inhibition constant K_i_ of 2.1 ± 0.6 mM. Hence, a concentration of 250 μM MeSH was selected as the saturating, non-inhibitory substrate concentration in the final assay setup.

The assay demonstrated high sensitivity, with the H_2_O_2_/CBA detection being significantly more sensitive compared to H_2_S/AzMC. Specifically, quantification of H_2_O_2_ production via CBA yielded an LOD and LOQ of 0.1 and 0.29 μg enzyme/ml, respectively (Fig. 3I–Supplementary Table 3). In contrast, the H_2_S detection using AzMC showed an LOD and LOQ of 1.59 and 4.84 μg/ml, respectively (Fig. 3I–Supplementary Table 3). This establishes the H_2_O_2_ detection method as the primary, most sensitive readout for MTO activity. Higher sensitivity of the H_2_O_2_/CBA detection is not due to a higher rate constant of the detection reaction compared to the H_2_S/AzMC route (Supplementary Fig. 3), but likely rather due to other factors, such as lower background fluorescence of CBA, higher quantum yield of the CBA product as well as stability and volatility of the measured analyte. In addition, it was previously reported that H_2_S:AzMC stoichiometry is 2:1 (compared to 1:1 stoichiometry of H_2_O_2_:CBA), which could contribute to overall higher sensitivity of H_2_O_2_/CBA detection [20].

Lastly, we also compared our newly developed kinetic MTO assay with the current state-of-the-art coupled endpoint assay where MeSH is first generated by methionine γ-lyase (MGL), followed by discontinuous, endpoint detection of H_2_S using colorimetric lead acetate paper and H_2_O_2_ via the AmplexRed-HRP system (Fig. 3J) [19]. Our novel kinetic MTO assay demonstrated dramatically improved sensitivity over the established method. The new assay quantified H_2_S and H_2_O_2_ with LOQs of 4.84 and 0.29 μg/ml, respectively. This represents a 27-fold and 60-fold increase in sensitivity for H_2_S and H_2_O_2_, respectively, compared to the previous method (LOQ: 129.7 and 17.6 μg/ml) (Fig. 3K and L, Supplementary Table 4).

In summary, the developed assay represents a significant advancement over the previous methods, offering a direct, sensitive, and kinetically robust system to study MTO catalytic properties.

### 2.3 Quantification of MeSH in biological, dietary and environmental samples

To showcase the robustness and broad utility of our newly developed MeSH metabolism toolkit, we used it to quantify MeSH generation and degradation across a range of complex, real-world samples, from cellular models through a variety of foods to environmental systems.

**Fig. 4:**
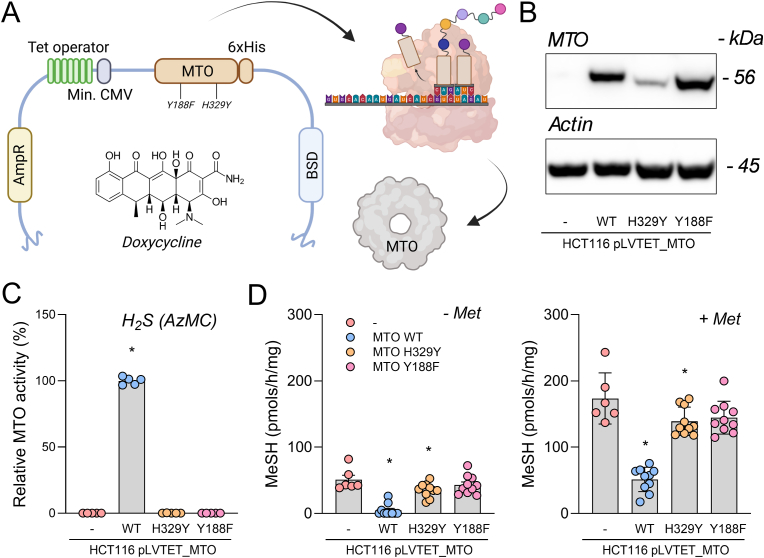
**Relationship between MTO and cellular methanethiol (MeSH) emissions**. **A** – Schematic depiction of the doxycycline-inducible MTO expression in stably modified HCT116 cells. **B** – Representative immunoblots illustrating expression of MTO variants in lysates of the modified HCT116 cells compared to the unmodified controls. **C** – Relative MTO activity measured using AzMC (H_2_S) in lysates from unmodified (control) and induced modified HCT116 cells (n = 5). **D** – MeSH production in the absence (left) and presence (right) of 1 mM methionine after 4 h incubation of the indicated HCT116 cell homogenates (n = 10). All data are presented as mean ± SEM and asterisk (*) indicates significance (*p* < 0.05).

Given the emerging roles of MeSH and MTO in cancer biology, we deployed our assays in a controlled cellular system. We genetically modified HCT116 colorectal cancer cells to stably express MTO from a doxycycline-inducible promoter (Fig. 4A). This system allowed fine-tuned, physiological level-mimicking expression of MTO including wild-type MTO (MTO WT) and two catalytically impaired missense variants MTO H329Y [19] and MTO Y188F (previously unpublished). The pathogenic H329Y mutant is likely inactive due to misfolding, which is also reflected in its decreased steady-state levels (Fig. 4B). In contrast, Y188F appears to be a catalytically dead mutant with proper folding and retained stability based on its steady-state levels (Fig. 4B). We confirmed that only the MTO WT-expressing HCT116 cells exhibited significant MTO activity, with no detectable MTO activity in untransduced control cells (Fig. 4C). The level of MTO activity correlated well with the amounts of MeSH in these cells (Fig. 4D). Specifically, unmodified HCT116 cells serving as control produced 50.0 ± 6.8 pmol/h/mg protein MeSH. In stark contrast, MTO WT-expressing cells showed a >90% reduction in MeSH production (4.6 ± 2.9 pmol/h/mg protein), demonstrating efficient intracellular clearance. On the other hand, HCT116 cells expressing catalytically impaired MTO H329Y and MTO Y188F variants showed even lower levels of MeSH compared to unmodified controls with 34.9 ± 3.4 and 42.9 ± 4.5 pmol/h/mg protein MeSH, respectively. Supplementation of lysates with 1 mM methionine increased methanethiol production approximately 3-4-fold across all groups, except for MTO WT cells, which exhibited a 10-fold increase, likely due to substrate saturation/inhibition of the enzyme (Fig. 4D). These results show a clear inverse relationship between MTO expression and net MeSH levels in a cell model and illustrate how catalytically competent MTO maintains low levels of MeSH.

**Fig. 5:**
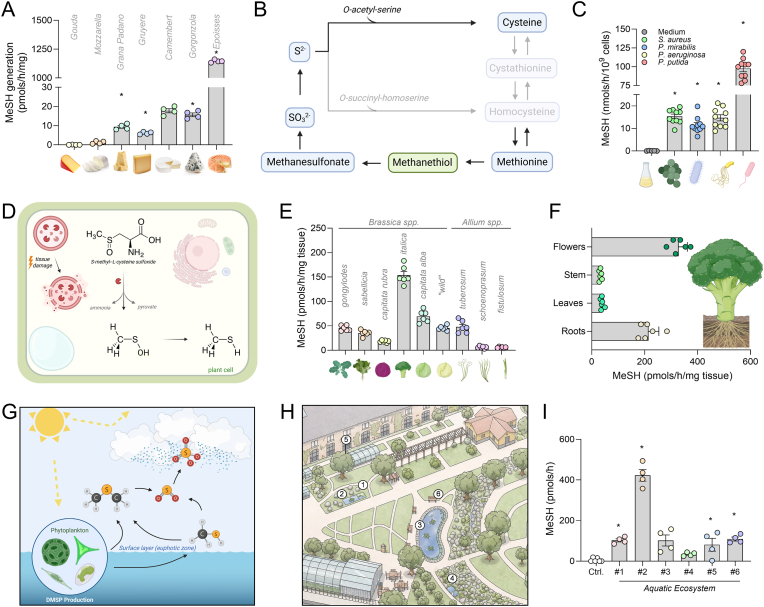
**Application of the MeSH assay toolkit across diverse biological systems. A** – Quantification of MeSH emission from various types of cheeses. Cheese samples were homogenized in HBS, and the resulting suspension was incubated for 2 h at 37°C. Thereafter, MeSH was volatilized, subjected to a phase transfer, trapped in the alkaline solution, and derivatized with DBD-F (n = 4). **B** – Schematics of the methionine catabolism pathway in *P*. *putida*, leading to increased MeSH production via MGL. **C** – Production of MeSH in bacterial species. Fresh LB medium was inoculated with the indicated bacterial cultures to an OD_600_ of 0.3. After 1 h of incubation at 37°C, MeSH was volatilized, subjected to a phase transfer, trapped, and derivatized with DBD-F (n = 10). **D** – Model of the “mustard oil bomb” defense mechanism in plants, where tissue damage (crushing) activates cystine lyase, which cleaves S-methyl-l-cysteine sulfoxide (SMCSO), generating MeSH and other VSCs. **E&F** – Assessment of MeSH content in leaf tissues of several *Brassica* and *Allium* species (**E**) and in different anatomical parts of broccoli (*Brassica italica*, **F**). Samples were homogenized using a cooled pestle and mortar as well as a bead mill. Following a 2-h incubation at 37°C, MeSH was volatilized, subjected to a phase transfer, trapped, and derivatized with DBD-F. For the tissue distribution analysis (**F**), broccoli (*Brassica italica*) was further dissected into distinct anatomical parts (e.g., roots, stems, leaves, flowers) prior to the MeSH assay (n = 6). **G** – Conceptual schematic of the CLAW hypothesis, illustrating the proposed feedback loop where marine biota influence climate by emitting VSCs including MeSH that form cloud condensation nuclei (DMSP = Dimethylsulfoniopropionate). **H&I** – The university botanical garden lentic aquatic systems (**H**) were sampled for evaluation of MeSH concentrations after 3-h incubation at 37°C. Thereafter, samples were subjected to volatilization, phase transfer, trapping, and derivatization with DBD-F (**I**; n = 4). All data are presented as mean ± SEM and asterisk (*) indicates significance (*p* < 0.05).

As MeSH is also a key flavor determinant in fermented foods, we assessed MeSH emissions in various cheese samples (Fig. 5A). Fresh cheese (e.g. Gouda, Mozzarella) showed minimal MeSH emissions (<1.2 pmol/h/mg), while longer-ripened hard cheeses (e.g. Grana Padano, Gruyère) emitted significantly more (6.1 and 9.4 pmol/h/mg, respectively). Soft, mold-ripened cheeses (e.g. Gorgonzola, Camembert) produced even higher amounts (15.6 - 17.7 pmol/h/mg, respectively) with the washed-rind cheese Époisses exhibiting an exceptionally high 1146 pmol/h/mg MeSH emissions, consistent with its potent aroma. Thus, our analysis revealed a clear correlation between cheese ripening and MeSH emissions.

Sulfur metabolism in bacteria also represents a significant source of MeSH emissions (Fig. 5B and C). Production of MeSH depends on the presence and activity of sulfur assimilating and converting pathways, specifically the transsulfuration pathway (Fig. 5B). Cultured *P*. *putida* showed significantly higher MeSH emissions of 98.0 pmol/h/10^9^ cells than the cultures of *S. aureus*, *P. mirabilis* and *P. aeruginosa* showing MeSH levels between 11.5 and 15.4 pmol/h/10^9^ cells (Fig. 5C).

In plant biology, MeSH mediates complex ecological interactions, including chemical defenses against tissue damage caused by various microbes or herbivores. Upon mechanical damage, MeSH is produced from S-methyl-l-cysteine sulfoxide (SMCSO) and released to prevent further damage in certain plant species (Fig. 5D). Our analysis showed that all tested *Brassica* species plants, namely *B. gongylodes* (kohlrabi), *B. sabellica* (kale), *B. capitata/capitata alba/capitata rubra* (wild/red/white cabbage) and *B. italica* (broccoli), produced MeSH upon mechanical damage with broccoli being the most potent producer (153 pmol/h/mg tissue; Fig. 5E). Detailed analysis of the broccoli plant localized the highest MeSH production to the roots and flowers (Fig. 5F), consistent with these parts being the most exposed to the surrounding environment. In comparison, of the *Allium* species plants tested, only *A. tuberosum* (garlic chives) showed detectable levels of MeSH, likely related to their known production of allicin and related sulfur compounds for similar purposes. This analysis highlights the specificity of sulfur metabolic pathways and sulfur-based chemical defenses in plants.

Lastly, we used our assay to evaluate samples from aquatic systems due to the role of VSCs in environmental science. As an example, Fig. 5G depicts the role of VSCs in cloud generation (the CLAW hypothesis). Therefore, we collected samples from different lentic ponds found in the University of Fribourg botanical garden (Fig. 5H). We found that all these water samples had significantly higher MeSH levels compared to tap water used as control (Fig. 5I). Sample Site 2 was identified as a particularly prominent MeSH source, confirming the sensitivity and utility of the method in analyzing samples of complex environmental matrices.

## 3 Discussion

The broad biological significance of MeSH has long been starkly disproportionate to the accessibility of analytical tools for its study. Its intrinsic properties, including high vapor pressure, reactivity, and low physiological concentrations, have confined its detailed investigations to laboratories equipped with sophisticated, expensive chromatographic systems. Here, we bridge this gap by introducing a versatile, fluorometric toolkit that makes the research on MeSH accessible to anyone interested. Our toolkit consists of two complementary methodologies: a highly sensitive and selective fluorometric MeSH quantification assay and a real-time kinetic MTO enzymatic assay. By leveraging standard laboratory equipment, these methods transform MeSH from an analytical challenge into a readily quantifiable analyte, thereby opening new avenues of research across fields.

The core achievement of our fluorometric MeSH quantification assay is its specificity and sensitivity comparable to the state-of-the-art gas chromatography-based methods. Specifically, the “tube-in-tube” design is a key innovation, enabling continuous and efficient capture of MeSH in a closed system (Fig. 2). This simple yet effective design circumvents the sample loss that plagues traditional endpoint headspace sampling, a critical advantage for a molecule as elusive as MeSH [13]. The assay's excellent sensitivity (LOD of 124 nM) and wide linear range make it suitable for the concentrations relevant to most biological systems (Fig. 2C). Furthermore, its high selectivity, demonstrated by the negligible interference from non-volatile thiols, H_2_S and structurally similar alkylmercaptans at physiological levels, ensures reliable quantification in complex matrices (Fig. 2E). High specificity of our MeSH quantification assay over other thiols or VSCs arises primarily from three factors: (i) the volatility of MeSH (determining whether it can undergo phase transfer from the sample into the headspace), (ii) its solubility in the alkaline trapping solution, and (iii) the fluorescence quantum yield of the resulting DBD-thiol adduct. The optional liberation of disulfide-bound MeSH using TCEP further enhances the assay's utility for studying total MeSH pools in biological samples, providing a complete picture compared to the methods measuring only the free volatile fraction (Fig. 2F). Therefore, we anticipate that such advantage will be particularly relevant for highly oxidized biological samples such as plasma or urine [7,21,22].

Building on this foundation, our real-time kinetic MTO enzymatic assay addresses critical limitations of the previously developed coupled MGL-MTO assay (Fig. 3J) [19]. The reliance on MGL to generate MeSH *in situ* introduced unnecessary complexity and potential sources of error. Our method, which utilizes pre-formed MeSH trapped in alkaline solution, provides a direct measurement of MTO activity. Furthermore, the ability to continuously and independently quantify both products of MTO-catalyzed degradation of MeSH, namely H_2_S and H_2_O_2_ (Fig. 3), represents another advantage as their stoichiometric production can vary between orthologs exemplified by the *Rhodobacteraceae* family MTO ortholog, which was described as predominantly generating sulfane sulfur rather than H_2_S [23]. Characterization of *Rhodobacteraceae* family MTO ortholog could also offer a possible explanation for the substrate inhibition of human MTO found in our study (Fig. 3H), which can be explained by binding to an unknown allosteric site and/or modifying the enzyme. Conserved cysteine residues of *Rhodobacteraceae* family MTO were found modified by MeSH and their mutagenesis analysis into serine confirmed their importance for catalysis and production of sulfane sulfur [23]. While Cu^2+^ concentrations below 5 μM did not have any significant impact on MTO activity (also indicating full saturation of the purified MTO with the cofactor; Fig. 3F), higher concentrations resulted in a declining signal. This could have been due to direct disruption to enzymatic function (e.g. redox cycling, oxidative damage or unspecific binding of Cu^2+^ ions) or to the probe detection reaction (e.g. MeSH/H_2_S scavenging by excess copper) [24].

The applications of our toolkit are numerous and highly relevant to biomedical, biological and environmental research as exemplified in Fig. 4, Fig. 5. MeSH has recently emerged as a potential volatile biomarker in oncology [1,25]. We previously postulated that its accumulation in tumors results from both increased production through altered methionine metabolism and impaired elimination due to the frequent downregulation of MTO [1]. Expression of MTO inhibits the progression of colorectal cancer by suppressing epithelial-mesenchymal transition, a key step towards the aggressive spread of cancer in the human body [25]. Therefore, MTO is considered a tumor-suppressor and monitoring its status (expression, polymorphism) and activity could present an important and interesting target in cancer research. Furthermore, mutations in MTO causing reduced MTO activity result in extraoral halitosis and constitute the genetic background for a recently identified inborn error of sulfur metabolism [8].

Our assays also provided a quantitative fingerprint of MeSH production that perfectly aligned with sensory expectations from different types of food. The spectrum of its emissions, from trace levels in fresh cheeses to very high levels in Époisses cheese, provided chemical justification for aroma profiles traditionally described qualitatively (Fig. 5A). Although MeSH is just one of many VSCs in cheese, its role as a key flavor determinant is well-established [26].

The identification of *P. putida* as a high MeSH producer provides a clear biochemical explanation for how the absence of the reverse transsulfuration pathway redirects methionine catabolism towards the generation of VSCs [27]. Specifically, the presence of a functional reverse transsulfuration pathway in *P. aeruginosa* facilitates the direct conversion of methionine to cysteine, conserving sulfur within the cellular metabolite pool. In contrast, the absence of this pathway in *P. putida* requires an alternative catabolic route involving MGL, which results in the obligatory production of MeSH leading to increased emissions of this volatile intermediate in *P. putida* (Fig. 5C) [27]. Notably, a similar metabolic rerouting was observed in individuals suffering from classical homocystinuria, an inborn error of sulfur amino acid metabolism caused by a lack of cystathionine beta-synthase (CBS) activity [28]. Inactivation of CBS by pathogenic mutations blocks the reverse transsulfuration pathway, which leads to accumulation of homocysteine, a substrate of CBS, and methionine as a sole metabolic source of homocysteine. High levels of methionine are funneled through the alternative catabolic routes that result in the accumulation of MeSH [7].

In plant biology, we captured the dynamics of the so-called mustard oil bomb (formerly known as the glucosinolate-myrosinase complex), a plant defense mechanism against herbivores found in members of the *Brassicaceae* family [29]. MeSH in mustard oil bomb is not derived from glucosinolates, but rather originates from the enzymatic degradation of SMCSO, a major sulfur-containing metabolite in *Brassicaceae* family [4,30,31]. Upon tissue damage, cystine lyase cleaves SMCSO, yielding methanesulfenic acid, which is a key precursor for various VSCs including MeSH and DMDS (Fig. 5D). Beyond its function in plant chemical defense, MeSH also serves a role in ecological signaling. Using MeSH as a precursor, flowers of the *Aristolochiaceae* family produce DMDS creating an olfactory mimicry of rotting flesh to attract saprophilous pollinators [5]. This biosynthetic pathway is initiated by MGL, which cleaves methionine to generate MeSH. Subsequently, a specialized enzyme disulfide synthase, a convergently evolved enzyme derived from the MTO family, oxidizes the generated MeSH into the volatile attractant DMDS. Taken together, our assay toolkit represents a robust tool to dissect the dual roles of MeSH metabolism in both plant defense and pollinator attraction.

Lastly, application of our toolkit to natural aquatic environments underscores its utility beyond controlled and reductionist studies in biomedical and biological fields to complex ecosystems in environmental science. Recently renewed focus on marine sulfur cycling posits that marine biota (phytoplankton) regulate climate by emitting VSCs that form sunlight-scattering aerosols (the CLAW hypothesis; Fig. 5G) [11,32]. A recent modeling demonstrated that MeSH is a significant, yet overlooked, source of sulfur dioxide (SO_2_) contributing to its regional surface layer concentration increases of up to 40–60% [12]. Detecting meaningful variations in MeSH levels across different pond samples (Fig. 5I) validated our assay's sensitivity and suitability for quantifying and monitoring this underappreciated player for improvements in the modeled representation of sulfur spatiotemporal patterns relevant to air quality predictions and climate impact assessment.

While sensitive and sufficiently robust, our assay toolkit has specific limitations. The MTO assay requires separate measurements for H_2_S and H_2_O_2_ due to a spectral overlap of the used fluorescent probes. Because of relatively slow kinetics of these probes, samples exhibiting high MTO activity may be underestimated and thus would require pre-dilution to obtain a true MTO activity of the sample. As for every enzymatic assay, particularly when analyzing more complex biological samples using fluorescent probes, one should always consider the impact of non-enzymatic and enzymatic scavengers of the measured analyte (e.g. Cu^2+^ or rhodanese activity on H_2_S and catalase activity on H_2_O_2_) and changes in parameters affecting the detection reaction (e.g. pH or probe concentration). Analysis of highly complex samples containing competing VSCs, which could interfere with MeSH quantification, may result in a false overestimation and would require a standard chromatographic approach. However, this was not the case in our test of samples from *Allium* species, which produce longer-chain alkylmercaptans and other organosulfur secondary metabolites that did not yield meaningful interference (Fig. 5E) [33].

In conclusion, we have designed, developed and validated a comprehensive methodological solution to the long-standing analytical challenge of MeSH quantification. By combining high sensitivity and specificity with unparalleled accessibility, this fluorometric assay toolkit effectively bridges a critical gap in sulfur redox biology. We anticipate these assays will enable significant advancements across multiple fields. The MeSH assay can be deployed for real-time food freshness monitoring, studying plant-microbe interactions in the rhizosphere, and mapping global MeSH fluxes to better understand its environmental impact. Furthermore, the MTO assay can find applications in screening patient samples for inborn errors of metabolism, investigating the enzyme's role in cancer or studying its evolution and properties across all kingdoms of life. We are convinced that our MeSH metabolism toolkit will provide researchers with the means necessary to elucidate the multifaceted roles of MeSH and MTO in human health and to accelerate discoveries in a broad spectrum of scientific disciplines from microbiology and plant biology to food chemistry and environmental science.

## 4 Materials and methods

***Chemicals and enzymes.*** Unless stated otherwise, all chemicals were purchased from Sigma or Fisher Scientific.

***Cell culture.*** The HCT116 human colorectal carcinoma cell line was obtained from American Type Culture Collection (ATCC) and was cultured in a modified McCoy's 5A medium (Gibco, ThermoFisherScientific) supplemented with 10% fetal bovine serum (FBS; Gibco), 100 U/ml penicillin and 100 μg/ml streptomycin (Gibco).

Stable HCT116 cell lines expressing MTO WT, MTO H329Y and MTO Y188F variants were generated using lentiviral transduction as described previously [34]. Briefly, a codon-optimized sequence of human MTO with a C-terminal 6xHis tag was synthesized by Genscript and cloned into a pLVTET backbone (Addgene# 194072). Lentiviral particles were produced in HEK293T cells (ATCC). At 24 h post-transfection of HEK293T cells (ATCC), the supernatant containing lentiviral particles was harvested, filtered through a 0.45 μm filter, aliquoted, and stored at −80°C. Subsequently, HCT116 cells were transduced in the presence of 6 μg/ml protamine sulfate. Selection was initiated 72 h post-transduction by 12 μg/ml blasticidin in the medium. Cells were maintained under blasticidin selection for three passages to ensure the establishment of a stable polyclonal population. MTO expression was induced by treating cells with 2 μg/ml doxycycline. After 24 h, the cells were harvested, washed once with PBS via centrifugation (200 RCF, 5 min), and the pellets were stored at −80°C until analysis.

***Expression and purification of recombinant MTO.*** A codon-optimized gene sequence for MTO WT with a C-terminal 6xHis tag was synthesized by Genscript and cloned into a pET28a backbone (Sigma-Merck). The construct was transformed into *E. coli* BL21 Gold (DE3) competent cells (Agilent). For protein expression, a single transformed colony was used to inoculate a 5 mL LB starter culture, which was then expanded into 500 mL of LB medium containing 100 μg/ml ampicillin. The main culture was grown at 37°C with shaking at 200 rpm until the mid-log phase (OD_600_ ∼ 0.8). Expression was induced with 0.6 mM isopropyl β-d-1-thiogalactopyranoside (IPTG), and the culture was incubated for an additional 4 h at 22°C with shaking. Cells were harvested by centrifugation (9,000xg, 10 min, 4°C), and the pellet was washed with ice-cold phosphate-buffered saline (PBS) before storage at −80°C.

For purification, frozen bacteria pellets were thawed and resuspended in HEPES-buffered saline (HBS; 100 mM HEPES, 150 mM NaCl, pH 8.0). Lysis was performed by the addition of 1 mg/ml lysozyme and DNase I, followed by incubation for 30 min at 4°C with constant agitation and subsequent sonication on ice (8 cycles of 2 min, 50% duty cycle; Misonix S-3000). The soluble fraction was isolated by centrifugation (9,000xg, 10 min, 4°C). The clarified lysate was applied onto a 2 mL column containing 0.5 mL of cobalt-loaded immobilized metal affinity chromatography resin equilibrated in a Binding buffer (50 mM sodium phosphate, 300 mM NaCl, pH 7.4). The resin was washed with five column volumes of binding buffer, and the captured MTO was eluted with binding buffer supplemented with 200 mM imidazole. The eluate was buffer-exchanged into HBS and concentrated using a 0.5 mL Amicon centrifugal filter (Millipore). For Fig. 3K, a MTO construct containing a C-terminal Strep-tag was used and purified as previously described [19].

***Preparation of alkylmercaptan solutions.*** All procedures involving alkylmercaptans were conducted on ice within a well-ventilated fume hood to minimize volatility and mitigate risks associated with their intense odor and high toxicity. MeSH and ethanethiol (low molecular weight thiols) were obtained in their sodium salt forms, whereas propanethiol and butanethiol were acquired as liquids. Stock solutions (20–100 mM) were prepared in 0.5 M NaOH, which effectively suppresses volatility. These alkaline stock solutions are stable, tolerate multiple freeze-thaw cycles, significantly reduce odor, and enhance overall safety for routine handling.

***Generation of methanethiolated BSA.*** Methanethiolated BSA was prepared via an oxidative conjugation process. The reaction of 80 mg/ml BSA solution in HBS with mixed in 100 μM sodium methanethiolate (CH_3_SNa) was initiated by the addition of 1 mM H_2_O_2_. In this reaction, H_2_O_2_ oxidizes the free thiol of Cys34 residue to a reactive sulfenic acid, which then conjugates with the methanethiol nucleophile. The specified order of reagents is essential to ensure MeSH is present before H_2_O_2_-mediated oxidation, thereby minimizing competitive over-oxidation of the sulfenic acid to irreversible sulfinic and sulfonic derivatives.

***Fluorometric MeSH quantification assay*.** MeSH was quantified using a fluorometric assay based on volatilization and derivatization with DBD-F. The derivatization reagent was prepared from a 10 mM DBD-F stock solution in acetonitrile, which was stored at 4°C. A 20 μM DBD-F derivatization solution in 1% acetonitrile was prepared fresh daily. The analysis utilized a “tube-in-a-tube” configuration, wherein a PCR tube filled with 100 μl of 100 mM NaOH served as a VSC trap inside a sealed 1.5 mL microcentrifuge tube containing 100-200 μl of sample or standard. Following an initial incubation of 1-4 h at 37°C, allowing for MeSH production, 100 μl of 20 mM TCEP in 100 mM HCl was added to the sample/standard to reduce disulfide bonds and liberate protein-bound MeSH. After an additional 1 h at 37°C incubation to permit VSCs to equilibrate into the headspace and be captured by the VSC trap, 100 μl of the alkaline trap solution was combined with 100 μl of the derivatization solution in a black 96-well plate. After vortexing and a 10 min incubation at RT, the fluorescence of the derivatized product was measured using a microplate reader at excitation/emission wavelengths of 390nm/526 nm.

For MeSH quantification in cultured cells, frozen cell pellets were thawed and resuspended in a minimal volume of CellLytic M buffer (Sigma), followed by a 10 min incubation on ice. The resulting homogenate was diluted to a final protein concentration of 12.5 mg/ml and a 200 μl aliquot served as a sample for the 4 h assay.

For MeSH determination in cheese, cheese samples were obtained from a local supermarket. For each cheese type, four distinct samples were obtained. For soft cheeses (Camembert and Époisses), each sample contained an equal mixture of rind and inner paste. All samples were weighed and homogenized in PBS using a Dounce tissue homogenizer to a final concentration of 125 mg/ml. A 200 μl aliquot of such homogenate served as a sample for the 2 h assay.

For MeSH quantification in bacteria, five individual colonies for each strain were selected from an agar plate and inoculated into 2 mL of Luria-Bertani (LB) medium and then grown overnight. The next morning the cultures were diluted with fresh LB medium to an OD_600_ = 0.3. A 200 μl aliquot of this suspension served as a sample for the 1 h assay.

For MeSH quantification in plant tissues, samples of *Brassica* and *Allium* species were procured from local supermarkets. From each, six leaf samples were collected. Broccoli (*Brassica italica*) was further dissected into distinct floral, leaf, stem, and root tissues. All samples were measured immediately after preparation. Tissues were first pulverized in a mortar and pestle pre-chilled with dry ice. The resulting powder was subsequently homogenized in a 1:4 (w/v) ratio with PBS in a 2 mL tube using a bead mill homogenizer (FisherScientific). Homogenization was performed with three 2.4 mm metal beads (Omni International) at a speed of 4 m/s for 240 s. A 100 μl aliquot of the clarified homogenate served as a sample for the 2 h assay.

For MeSH determination in lentic aquatic systems, water samples from various locations within the University of Fribourg botanical garden were carefully aspirated from the sediment-water interface using a Pasteur pipette while avoiding particulate matter or benthic sediment. Tap water was collected in parallel to serve as a control. A 200 μl aliquot of such sample served as a sample for the 3 h assay.

***Real-time kinetic MTO enzymatic assay*.** MTO enzymatic activity was determined by quantifying the production of H_2_S and H_2_O_2_ from its substrate MeSH using the fluorogenic probe 7-azido-4-methylcoumarin (AzMC) and coumarin boronic acid (CBA), respectively. Assays were carried out in black, clear-bottomed 384-well plates. Cell pellets were resuspended in a minimal volume of CellLytic M buffer, incubated on ice for 10 min and centrifuged at 12,000xg for 10 min at 4°C. The resulting cleared lysate was adjusted to a concentration of 5 mg/ml in HBS. For each well, 40 μl of such lysate solution was mixed with 38 μl of HBS containing 21 μM AzMC/CBA. The reaction was initiated by adding 2 μl of 10 mM MeSH solution (in 0.5 M NaOH), yielding a final substrate concentration of 250 μM in the 80 μl total reaction volume. The reaction mixture was homogenized by pipetting, the plate was sealed with an optical PCR foil, and fluorescence (excitation/emission: 365nm/450 nm AzMC and 332nm/470 nm CBA) was measured kinetically from the bottom of the plate for 2 h in 5 min intervals at 37°C without shaking. Control wells contained no MeSH substrate (i.e. received 2 μl of 0.5 M NaOH in lieu of MeSH). The MTO activity was reported as the background-corrected slope from the linear phase of the reaction. Specifically, the mean slope around the inflection point was calculated for each sample, subtracted from the value of the substrate-free control, and normalized to a designated control sample.

***MeSH detection using GC-SCD*.** The method was performed as described previously[35]. Briefly, MeSH calibration standards were generated using a CH_3_SNa stock solution at a concentration of 809 mg/l in Milli-Q water. From this stock, an intermediate solution containing 1.4 mg/l was prepared. The calibration curve for MeSH was carried out by adding the required volumes of this intermediate solution to the final solutions, covering a concentration range between 0.5 and 24 μg/l, with five concentration levels prepared in duplicate. The analysis of MeSH was carried out as was validated by Ontanon et al.[35]: 12 ml of the final solution and 40 μl of the internal standard solution (10 mg/l ethyl methyl sulfide (EMS) in ethanol) were added to a 20 ml vial.

The samples were analyzed by an Agilent 7890B gas chromatograph with a Sulfur Chemiluminescence Detector 8355 (GC-SCD) and a Supelco SPB-1SULFUR column (30 m × 0.32 mm I.D. × 4 μm film thickness), preceded by a 60 cm × 0.32 mm I.D. fused silica precolumn with polar deactivation. The precolumn crosses inside the Cryogenic Trapping System (CTS 2, Gerstel). The injection was performed with a Combi-PAL autosampler from CTC Analytics (Zwingen, Switzerland) in a multimode injector (MMI) at 150°C equipped with an ultra-inert liner of 1 mm I.D. After the injection, the syringe was purged with nitrogen for 5 min. The chromatographic oven was held at 35°C for 3.8 min then heated to 160°C at 10 °C/min and held at this temperature for 0.5 min. The carrier gas was He at a constant flow rate of 2 ml/min. The temperature program of CTS 2 was as follows: −150°C for 0.8 min and then raised at 20 °C/s up to 300°C. The detector temperature was 280°C, and the burner temperature was 800°C. The oxidizing gas flow rate was 50 ml/min, and the hydrogen flow rate was 38 ml/min in the “upper flow” and 7 ml/min in the “lower flow”.

**Identification of DBD-SCH_3_ by ^1^H NMR.** DBD-F (5.2 mg, 21.2 mmol) was dissolved in 1 ml of acetonitrile. A suspension of sodium methylthiolate (10.5 mg, 149 mmol) in 0.5 ml of acetonitrile was added and the mixture was stirred at room temperature for 30 min. The solvent was evaporated and the residue was placed at the top of a silica gel pad (2 cm*0.5 cm) using a minimal amount of chloroform. Flushing with 2 ml of chloroform gave a yellow oil (5.4 mg) containing a 1:2 mixture of unreacted DBD-F (**1**, 34%) and a product (**2**, 62%) that was identified as 4-(N,N-dimethylaminosulfonyl)-7-methylsulfanyl-2,1,3-benzoxadiazole (DBD-SCH_3_) matching the published data 36]: ^1^H NMR (400 MHz): 2.97 (6H, s), 7.18 (1H, dd, J = 8.68, 7.70 Hz), 8.05 (1H, dd, J = 7.70, 4.22 Hz). 2: 1H NMR (400 MHz): δ 2.72 (3H, s), 2.94 (6H, s), 7.06 (1H, d, J = 7.40 Hz), 7.93 (1H, d, J = 7.40 Hz).

***Statistics and data analysis.*** Data are presented as mean ± standard errors of the mean (SEMs). The number of replicates is indicated in the figure legends. Statistical analysis was typically performed using one-way ANOVA followed by Dunnett's multiple-comparison test. Differences with the p < 0.05 were considered significant and indicated in figures by asterisks. Plotting, curve-fitting and statistical analysis were performed using GraphPad Prism. Graphical design was done with BioRender.

## Data and materials availability

All data supporting the findings of this study are available in the paper and its supplementary information, if applicable.

## CRediT authorship contribution statement

**Thilo M. Philipp:** Conceptualization, Formal analysis, Investigation, Methodology, Visualization, Writing – original draft, Writing – review & editing. **Belen Schiffmann:** Investigation, Writing – review & editing. **Lucie K. Tintrop:** Investigation, Writing – review & editing. **Jacqueline Findlay:** Investigation, Writing – review & editing. **Niklas Krafczyk:** Investigation, Writing – review & editing. **Hanna Schlemminger:** Investigation, Writing – review & editing. **Susana Ainsa-Zazurca:** Investigation, Writing – review & editing. **Vicente Ferreira:** Methodology, Supervision, Writing – review & editing. **Pascal Fuchsmann:** Methodology, Supervision, Writing – review & editing. **Patrice Nordmann:** Conceptualization, Supervision, Writing – review & editing. **Lars-Oliver Klotz:** Conceptualization, Supervision, Writing – review & editing. **Csaba Szabo:** Conceptualization, Supervision, Writing – review & editing. **Tomas Majtan:** Conceptualization, Funding acquisition, Project administration, Supervision, Writing – original draft, Writing – review & editing.

## Declaration of competing interest

The authors declare the following financial interests/personal relationships which may be considered as potential competing interests: Tomas Majtan reports financial support was provided by Swiss National Science Foundation. Tomas Majtan reports a relationship with Travere Therapeutics Inc that includes: funding grants. If there are other authors, they declare that they have no known competing financial interests or personal relationships that could have appeared to influence the work reported in this paper.

## Data Availability

Data will be made available on request.
