# Supplementary material for: Fluorometric toolkit for methanethiol quantification and methanethiol oxidase activity determination in biological systems
Source: Redox Biol. 2026 Jul 8;95:104288. doi: 10.1016/j.redox.2026.104288 (PMC13380501; doi:10.1016/j.redox.2026.104288)

**Supplementary table 1. Raw source data for methanethiol (MeSH) quantification using the fluorometric DBD-F assay shown in Figure 2C.**

|  | **MeSH DBD-F fluorometric detection (AU)** | | | | | | | |
| --- | --- | --- | --- | --- | --- | --- | --- | --- |
| **MeSH**  **(µM)** | **Replicate 1** | **Replicate 2** | **Replicate 3** | **Replicate 4** | **Replicate 5** | **Replicate 6** | **Replicate 7** | **Replicate 8** |
| **0** | 1.5 | 1.6 | 1.4 | 1.8 | 1.4 | 1.4 | 1.4 | 1.6 |
| **0.0390625** | 1.7 | 1.8 | 1.7 | 2.0 | 1.8 | 1.9 | 2.2 | 1.9 |
| **0.078125** | 2.0 | 2.2 | 2.0 | 2.6 | 2.5 | 2.2 | 2.6 | 2.5 |
| **0.15625** | 3.1 | 3.4 | 3.4 | 3.6 | 4.0 | 4.1 | 4.2 | 4.5 |
| **0.3125** | 5.2 | 5.2 | 5.8 | 6.0 | 8.2 | 5.6 | 8.6 | 5.9 |
| **0.625** | 9.4 | 9.7 | 9.6 | 11.0 | 12.6 | 15.4 | 13.3 | 16.4 |
| **1.25** | 18.5 | 19.4 | 20.4 | 21.7 | 25.3 | 18.1 | 27.2 | 20.4 |
| **2.5** | 31.2 | 33.5 | 35.1 | 38.1 | 40.6 | 30.8 | 43.1 | 33.7 |
| **5** | 64.4 | 63.0 | 71.2 | 70.8 | 66.9 | 68.5 | 71.0 | 74.0 |
| **10** | 101.5 | 105.6 | 113.0 | 120.1 | 114.0 | 112.8 | 120.3 | 120.1 |

**Supplementary table 2. Raw source data for methanethiol (MeSH) quantification using the gas chromatography with sulfur chemiluminescence detection (GC-SCD) shown in Figure 2C.**

|  | **MeSH GC-SCD detection (AUC)** | |
| --- | --- | --- |
| **MeSH (µM)** | **Replicate 1** | **Replicate 2** |
| **0** | 0 | 0 |
| **0.0307** | 0 | 0 |
| **0.0614** | 14.8 | 15.8 |
| **0.1228** | 29.3 | 30.3 |
| **0.2456** | 47.5 | 46.5 |
| **0.4912** | 100.0 | 90.9 |

**Supplementary table 3. Raw source data for MTO activity assessment using our real-time kinetic MTO assay shown in Figure 3I.**

|  | **H_2_S (AzMC)** | | | | **H_2_O_2_ (CBA)** | | |
| --- | --- | --- | --- | --- | --- | --- | --- |
| **MTO**  **(µg/ml)** | **Replicate 1** | **Replicate 2** | **Replicate 3** | **Replicate 4** | **Replicate 1** | **Replicate 2** | **Replicate 3** |
| **0** | 0 | 0 | 0 | 0 | 12.02 | 12.32 | 13.73 |
| **0.05** |  |  |  |  | 11.41 | 12.18 | 11.76 |
| **0.0976563** |  |  |  |  | 15.27 | 15.38 | 15.76 |
| **0.1953125** | 9.42 | 3.99 | 0.00 | 0.00 | 18.34 | 18.05 | 20.16 |
| **0.390625** | 8.87 | 10.99 | 9.05 | 6.50 | 30.58 | 30.32 | 28.99 |
| **0.78125** | 9.02 | 18.93 | 4.68 | 15.75 | 45.31 | 44.66 | 45.49 |
| **1.5625** | 30.72 | 30.66 | 26.83 | 23.96 | 68.84 | 68.93 | 81.77 |
| **3.125** | 40.85 | 46.05 | 44.50 | 42.53 | 99.45 | 96.65 | 103.90 |
| **6.25** | 65.77 | 70.01 | 69.02 | 69.50 |  |  |  |
| **12.5** | 93.76 | 96.15 | 104.01 | 106.09 |  |  |  |

**Supplementary table 4. Raw source data for MTO activity assessment using coupled MGL-MTO assay shown in Figure 3L.**

|  | **H_2_S (PbAc)** | | | **H_2_O_2_ (AmplexRed-HRP)** | | |
| --- | --- | --- | --- | --- | --- | --- |
| **MTO**  **(µg/ml)** | **Replicate 1** | **Replicate 2** | **Replicate 3** | **Replicate 1** | **Replicate 2** | **Replicate 3** |
| **0** | 1.45 | 2.42 | 3.31 | 3.73 | 3.18 | 6.11 |
| **7.8125** | 2.23 | 3.32 | 2.02 | 15.95 | 14.23 | 20.84 |
| **15.625** | 1.07 | 2.96 | 2.04 | 35.81 | 35.32 | 36.24 |
| **31.25** | 4.52 | 6.79 | 6.76 | 49.37 | 61.58 | 62.72 |
| **62.5** | 11.83 | 25.35 | 15.46 | 79.41 | 129.06 | 91.54 |
| **125** | 22.96 | 50.92 | 37.70 |  |  |  |
| **250** | 45.63 | 77.74 | 65.41 |  |  |  |
| **500** | 89.68 | 105.87 | 104.45 |  |  |  |

**Supplementary figure 1. Identification of DBD-SCH_3_ product.** The ¹H-NMR spectrum (400 MHz, CDCl₃) of the derivatization probe DBD-F (**A**) and the reaction mixture of DBD-F with sodium methylthiolate (**B**). The spectrum shows a 1:2 mixture of unreacted DBD-F (1) and the product 4-methylthio-7-N,N-dimethylaminosulfonyl-2,1,3-benzoxadiazole (2). Key resonances for product (2): δ 2.72 (s, 3H, S–CH₃), 2.94 (s, 6H, N(CH₃)₂), 7.06 (d, J = 7.40 Hz, 1H, Ar–H), 7.93 (d, J = 7.40 Hz, 1H, Ar–H).

**
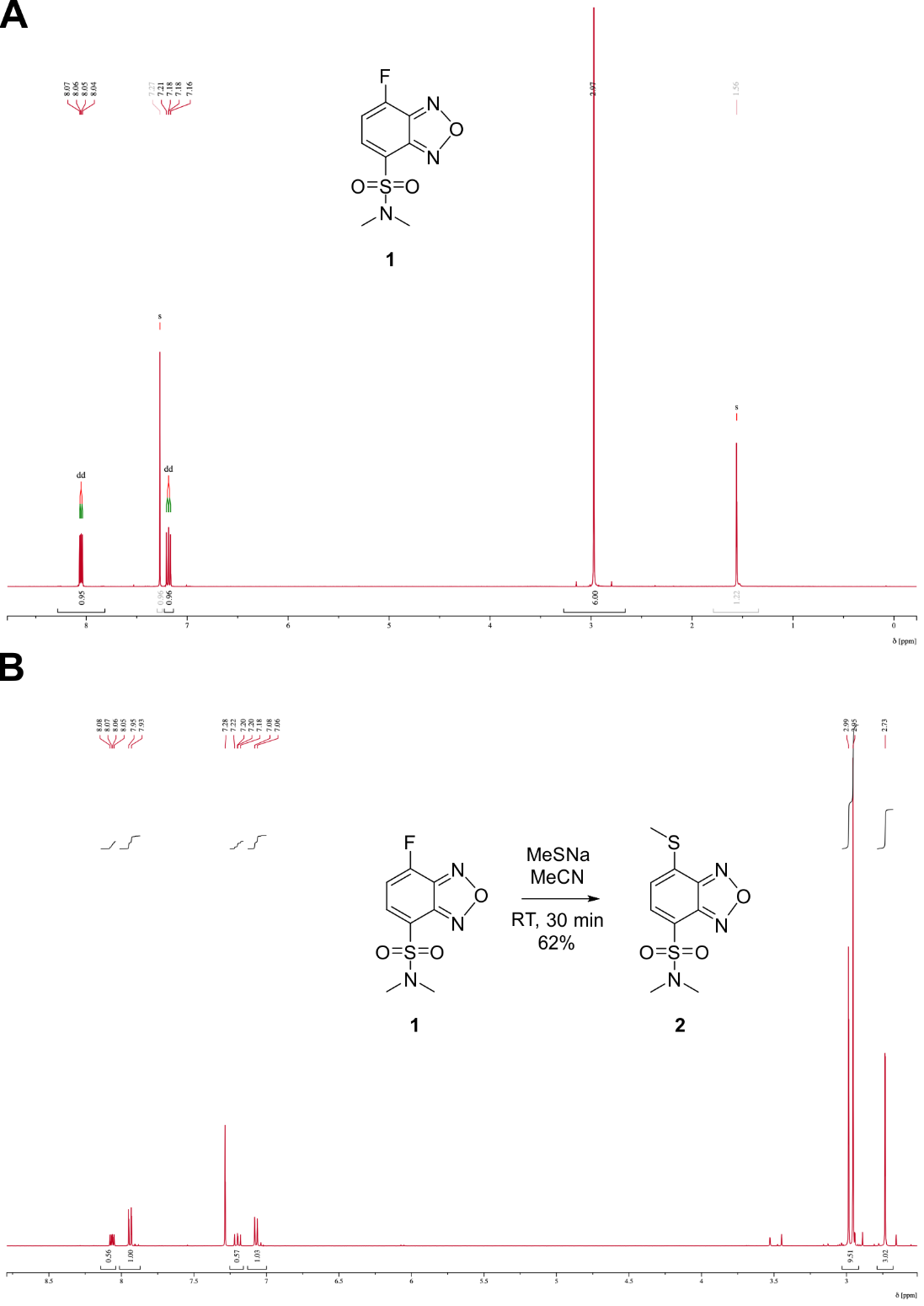
**

**Supplementary figure 2. Direct reaction of biothiols and alkylmercaptans with DBD-F probe.** Biothiols and alkylmercaptans (each at 10 µM) were directly reacted with DBD-F (20 µM) in solution for 10 minutes at room temperature (n=5). MeSH is highlighted in blue and used as a reference. Fluorescence was measured immediately thereafter at excitation/emission wavelengths of 390/526 nm. All data are presented as mean ± SEM and asterisk (*) indicates significance (p<0.05).


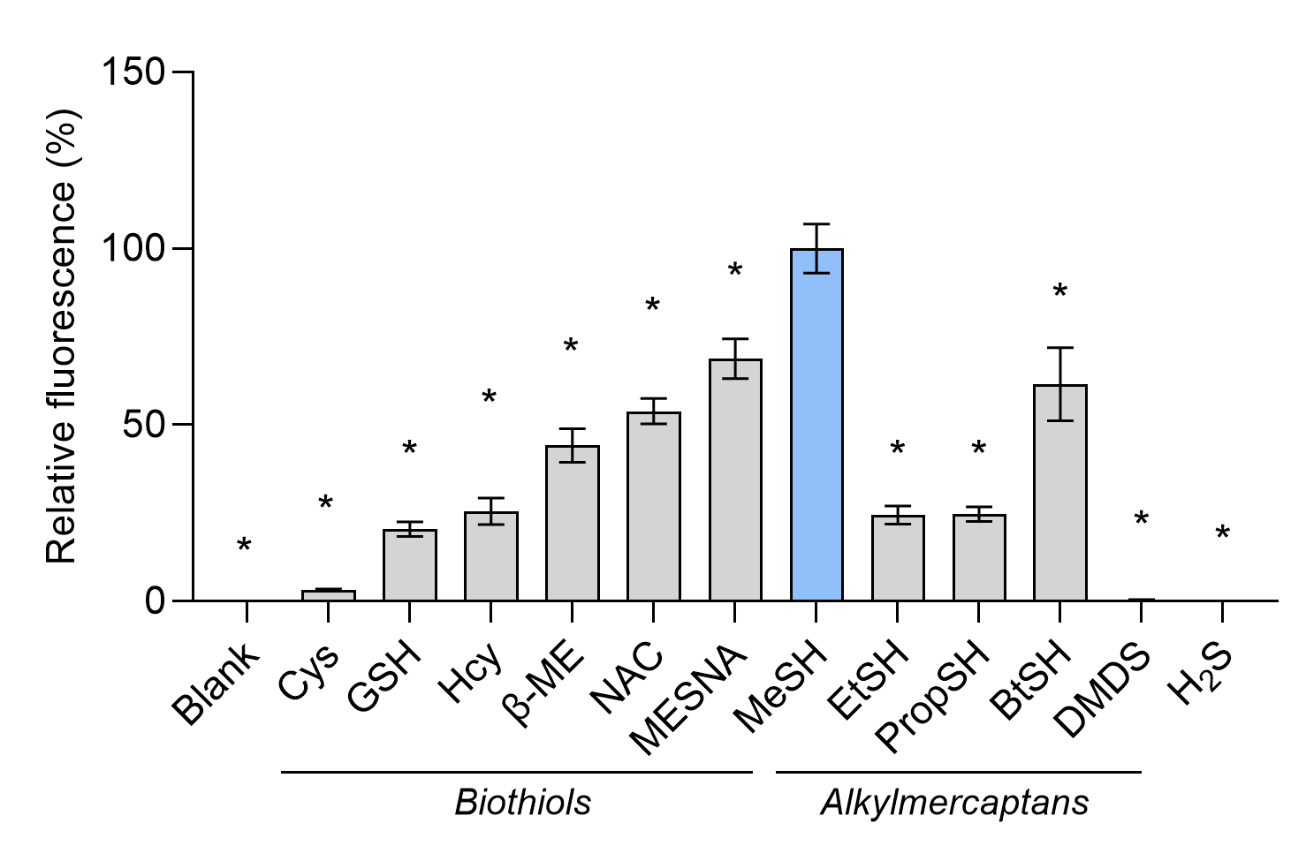


**Supplementary figure 3. Determination of detection rate constants of the fluorescence probes.** The respective substrates (100 µM MeSH for DBD-F (**A**), 100 µM H₂S for AzMC (**B**) and 100 µM H₂O₂ for CBA (**C**)) were directly reacted with each probe (10 µM DBD-F, 10 µM AzMC, or 10 µM CBA) in the respective assay buffers (DBD-F: 0.05 M NaOH, 0.5% ACN; AZMC/CBA: HBS). Fluorescence was monitored over time at the following excitation/emission wavelengths: 390/526 nm for DBD-F, 365/450 nm for AzMC, and 332/470 nm for CBA (n=6). All data are presented as mean ± SEM. The observed fluorescence increase was fitted to a pseudo-first-order reaction model to calculate the second-order rate constants (*k*) and midpoint of fluorescence change (t_1/2_).


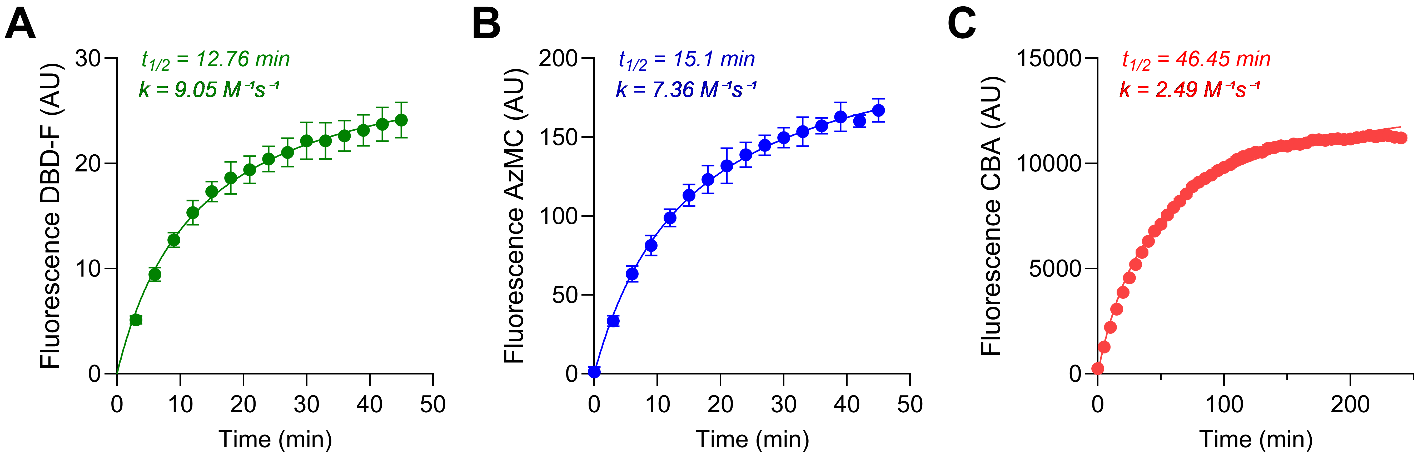

Supplement: Multimedia component 1 [file mmc1.docx]
